# Sarcopenia as a Predictor of Mortality in a Cohort of Elderly Patients Undergoing Endoscopic Retrograde Cholangiopancreatography

**DOI:** 10.3390/life15010021

**Published:** 2024-12-28

**Authors:** Giacomo Mulinacci, Clara Benedetta Conti, Alberto Savino, Davide Gandola, Davide Ippolito, Roberto Frego, Alessandro Ettore Redaelli, Marta Maino, Marco Emilio Dinelli

**Affiliations:** 1 Interventional Endoscopy Unit, Fondazione IRCCS San Gerardo dei Tintori, 20900 Monza, Italy; giacomo.mulinacci@irccs-sangerardo.it (G.M.);; 2 Department of Medicine and Surgery, University of Milano Bicocca, 20126 Milan, Italy; 3 Division of Radiology, Fondazione IRCCS San Gerardo dei Tintori, 20900 Monza, Italy

**Keywords:** sarcopenia, mortality, endoscopic retrograde cholangiopancreatography, skeletal muscle index, CT scan, elderly, ERCP, hepathobiliary endoscopy

## Abstract

Background and aims: Despite technical advances, endoscopic retrograde cholangiopancreatography (ERCP) is associated with complications and potentially lethal outcomes. Sarcopenia, a complex syndrome mainly associated with aging, has been recognized as a predictor of poor surgical outcomes. Thus far, the impact of sarcopenia on ERCP remains unknown. The present study evaluates the role of sarcopenia as a predictor of ERCP-related outcomes in a cohort of elderly patients. Methods: Patients who underwent ERCP between June 2019 and January 2023 were retrospectively included. Demographic and procedure-associated data were collected. Sarcopenia was assessed using the skeletal muscle index (SMI) measured from a single axial slice through the L3 vertebra on a CT scan. ERCP-related outcomes were recorded. Univariate and multivariate analyses were used to assess the correlation between sarcopenia and procedural outcomes. Results: In total, 256 patients were enrolled, of whom 30 (11.7%) were sarcopenic. Cardiopulmonary complications of ERCP occurred in 3.5%. Sarcopenia was associated with higher 30-day and 12-month post-ERCP mortality (OR 3.45, *p* = 0.03; OR 3.87, *p* = 0.004) and longer hospitalization time (7 vs. 11 days, *p* = 0.003). Conclusions: SMI is an easy and objective index of sarcopenia that could be used to predict ERCP outcomes. Indeed, sarcopenia was independently associated with prolonged hospitalization and increased mortality in a retrospective cohort of elderly patients.

## 1. Introduction

Endoscopic retrograde cholangiopancreatography (ERCP) is an invasive combined endoscopic and fluoroscopic procedure for the treatment of biliary and pancreatic benign and malignant diseases [1]. Indeed, ERCP is employed in a variety of contexts, including bile stones removal, drainage of obstructed ducts, relieving obstructions, managing strictures, treating leaks, and performing biopsies to evaluate malignancies.

ERCP is associated with significant clinical complications, with a reported mortality of 1.2% [2,3]. The most common ERCP-related complications were defined by Cotton et al. and include pancreatitis, bleeding, infection, and perforation [4]. According to the largest studies, the frequency of ERCP-related complications varies from 7% to 12% [5,6]. Of them, pancreatitis is the most frequent, with a reported prevalence of 3.5–4.9% [5,7]. When compared to the complications defined by Cotton et al., cardiopulmonary complications of ERCP are less documented in the existing literature, mostly due to the high heterogeneity in their definition [8]. Accordingly, their reported prevalence varies widely, reaching 2.4% in larger studies [8].

The American Society for Gastrointestinal Endoscopy (ASGE) grading system and the American Society of Anesthesiologists’ (ASA) score are subjective assessments of a patient’s overall health based on five classes (I to V) [9,10]. They are among the most used systems to stratify patients before an invasive procedure and their role as outcome predictors has been investigated in several surgical and endoscopic procedures, including ERCP [6,11]. However, there is a need for an objective, easily available, cost-effective, and robust predictive marker of outcomes. Numerous studies conducted in diverse surgical settings have demonstrated that sarcopenia, a complex syndrome characterized by a progressive decline in muscle mass, strength, and function [12], negatively impacts on surgical outcomes including post-operative complications as well as, short- and long-term mortality [13,14,15,16].

Sarcopenia mostly occurs in elderly patients secondary to the muscle ageing process (primary sarcopenia), although evidence of early sarcopenia in patients with chronic diseases (secondary sarcopenia) has been described [17,18]. Gold standard diagnostic tools for sarcopenia assessment include cross-sectional imaging techniques as Magnetic Resonance Imaging (MRI) and Computed Tomography (CT) scans. In particular, CT-based assessment of the skeletal muscle index (SMI) was proposed for the diagnosis of sarcopenia.

These advanced imaging modalities not only provide detailed visualization of muscle structures but also enable precise quantification of muscle mass, which is critical for sarcopenia diagnosis. Furthermore, CT scans are often performed for other clinical indications, making them a convenient and readily available tool for concurrent sarcopenia assessment.

Thus far, the role of sarcopenia in ERCP is still obscure. We conducted a retrospective study to assess the association of sarcopenia with ERCP-related outcomes in an Italian cohort of elderly patients.

## 2. Methods

We retrospectively searched the electronic health records of patients older than 75 years undergoing ERCP procedures for any diseases between June 2019 and January 2023 at IRCCS Fondazione San Gerardo Dei Tintori. The age cutoff to define “elderly” was that proposed by the “Italian Society of Gerontology and Geriatrics (SIGG)” [19]. The diagnosis period was selected to capture longitudinal data for at least 12 months. To be enrolled in the study, a patient was required to have undergone a CT-scan evaluation within 3 months from ERCP. The exclusion criteria were as follows: (1) failure of the ERCP procedure; (2) absence of records of patient height at the time of ERCP.

### 2.1. CT-Based Assessment of Sarcopenia-Related Indices

Two experienced radiologists (DG and DI), who were blinded to clinical and laboratory parameters, evaluated the CT images. Each scan was performed using two different 256-slice scanner (iCT Brilliance, Philips Healthcare, Hong Kong and iCT Elite, Philips Healthcare, Hong Kong), with a tube voltage of 120 kV and automatic tube current modulation. Raw data were reconstructed with a hybrid-iterative reconstruction algorithm (iDose4) if acquired on the iCT Brilliance or with an iterative model-based algorithm (IMR) if acquired on the iCT Elite. All the examinations were transferred to an image workstation (Philips BrillianceWorkspace, Philips Healthcare, Hong Kong) to select a single axial slice through the middle of the L3 vertebra and saved in DICOM format for the following analysis. The analysis was performed with the open-source image analysis software ImageJ (developed by the National Institutes of Health, ImageJ version 1.54), which gives results comparable to those of other software for body composition analysis, as previously described by van Vugt et al. [20]. Waist circumference was calculated after the scan by using pixel density thresholds (between −190 and −30 Hounsfield-Units) to draw regions of interest (ROIs) at the level of the abdominal wall and inside the abdominal muscular profiles. Skeletal muscle area (SMA), which included psoas, erector spinae, quadratus lumborum, transversus abdominis, external and internal oblique, and rectus abdominis muscles, was calculated using pixel density thresholds (between −29 and + 150 HU) and the ROIs already placed inside and outside the abdominal muscular profiles. The SMI was calculated using the following formula: SMI = SMA (cm^2^)/height^2^ (m^2^) [21]. Sarcopenia was defined as an SMI below the sex- and BMI- specific first quartile, according to what was proposed by van der Werf et al. [22].

### 2.2. Data Collection

Patient demographic and clinical data obtained before ERCP included age; sex; height; weight; BMI; and American Society of Anesthesiologists (ASA) score, a subjective assessment of a patient’s overall health that is based on five classes (I to V) [9]. ERCP data included procedure complexity according to the ASGE working party [10], and presence of hepatobiliary and/or pancreatic cancer.

Procedural outcomes included the following: (1) early (30 days) and medium-term (12 months) post ERCP mortality; (2) ERCP-related overall complications, including those proposed by Cotton et al. [4] together with cardiopulmonary complications, which we defined as any alteration of the cardiovascular and/or pulmonary status occurring within 48 h after the procedure and requiring a therapeutic approach; (3) duration of post-ERCP hospital stay.

### 2.3. Statistical Analysis

The statistical analysis was conducted using MedCALC (version 23.0.9) and STATA software (version 18.5). Continuous data were compared using the independent-samples *t*-test or the Mann–Whitney U test. For categorical data, the chi-square test or Fisher’s exact test was used. To identify potential prognostic factors for ERCP outcomes and survival, univariate analyses were performed first, and a multivariate analysis was further conducted using the logistic regression including all variables with *p* values of less than 0.05 in the univariate analysis, and those of clinical interest. The discriminative performance of the models was evaluated by measuring the area under the receiver operating characteristic (AUROC).

If the *p* value was less than 0.05, the differencewas considered statistically significant.

## 3. Results

Among 650 consecutive patients older than 75 years who underwent ERCP from June 2019 to January 2023, 256 met the inclusion criteria and were included in this study. The prevalence of sarcopenia in the study cohort was 11.7%. The median time from CT-scan evaluation to ERCP was 3 days (IQR 0–9). Baseline demographic patient characteristics did not significantly differ between the sarcopenic and non-sarcopenic cohort (Table 1).

ERCP difficulty, measured through the ASGE grading system criteria [10], did not significantly differ between sarcopenic and non-sarcopenic patients (10% vs. 15.9%, *p* = 0.39). The indications for ERCP are represented in Appendix A Table A1.

### 3.1. ERCP Complications and Outcomes

None of the patients died during the procedure. ERCP-related complications occurred in 47 (18.3%) procedures. In particular, thirty-eight (14.8%) were of the types defined by Cotton et al. [4], while cardiopulmonary complications were 9 (3.5%). As for the former, pancreatitis and bleeding represented most of the complications, occurring in 28 patients (73.7%). Cardiovascular and pulmonary complications were almost equally distributed, with four (44.4%) and five (55.6%) instances, respectively.

### 3.2. ERCP Complications and Outcomes: Sarcopenic vs. Non-Sarcopenic Patients

The 30-days and one-year mortality rates were significantly higher in the sarcopenic group than in the non-sarcopenic group (6.2% vs. 23.3%, *p* = 0.001; 13.7% vs. 40%, *p* = 0.003). The indications for ERCP in sarcopenic patients’ death within 30 days from the procedure included cholelithiasis for 2 patients, pancreatic cancer for 2 patients, and acute pancreatitis for 1 patient.

Analogously, the median duration of post-ERCP hospitalization was higher among sarcopenic patients (7 vs. 11 days, *p* = 0.003). In contrast, the frequency of overall complications did not differ between sarcopenic and non-sarcopenic patients (Table 2).

The risk factors for ERCP-related outcomes were then analyzed considering various background factors. In the univariate analysis, sarcopenia was the only parameter associated with an increased risk of short-term mortality (OR 4.61, *p* = 0.003). The significance was confirmed in the multivariate analysis adjusting for possible confounders (OR 3.45, *p* = 0.03, AUROC 0.77). By contrast, along with sarcopenia (OR 3.87, *p* = 0.004, AUROC 0.80), a higher ASA score and, CCI as well as the presence of cancer also correlated with 12-months mortality in the multivariate analysis (OR 3.18, *p* = 0.006; OR 1.36, *p* < 0.001; OR 1.84, *p* = 0.007). Finally, none of the assessed parameters had a significant impact on overall complications (Table 3).

Considering hepatobiliary cancer as a strong determinant of 1-year mortality, we also conducted a logistic regression analysis for the “non-cancer” group. Analogously to the previous cohort that included patients with cancer, the association of sarcopenia and 1-year mortality was confirmed (Supplementary Table S1).

## 4. Discussion

This monocentric, retrospective study showed that sarcopenia is associated with unfavorable outcomes in a cohort of Italian patients older than 75 years undergoing ERCP. Specifically, sarcopenia independently correlates with short-term (30 days) and medium-term (12-months) mortality, as well as with postoperative hospitalization duration.

The concept of frailty, defined by a person’s physiologic reserves and vulnerability to intrinsic and extrinsic stressors, has recently gained focus in surgical settings [23]. Indeed, robust data have shown the association of frailty with unfavorable surgical outcomes, including increased post-surgical complications and mortality [24]. However, the measurement of frailty largely depends on subjective clinical assessments, thus representing an important limitation for risk estimation in clinical practice. Sarcopenia has long been considered a major component of frailty, but the association between the two conditions has only recently been demonstrated [25]. In contrast to frailty, sarcopenia is objective, and measurable, as it can be easily diagnosed by evaluating the SMI via CT.

The negative impact of sarcopenia on surgical outcomes has been extensively described [13,14,15,16]. Possible mechanisms behind these findings include reduced amino acids delivery to the liver to produce acute-phase proteins and a lack of myokines-related IL-15 release hindering NK-cell development and survival and thus delaying post-surgical recovery [26,27]. Accordingly, the pre-operative assessment of sarcopenia might identify the patients at higher risk for surgical complications, lower short- and medium-term survival rates, and prolonged hospital stay [28,29,30]. Therefore, the concepts of pre- and post-operative rehabilitation, including exercise and nutritional interventions aimed at compensating for the reduced physical reserves occurring in the post-operative period [31,32]. Despite being generally less invasive than surgical interventions, ERCP can have clinically relevant complications.

The increased ERCP demand together with the population ageing led to a higher prevalence of elderly patients undergoing this procedure. The identification of a subset of patients at higher risk for ERCP negative outcomes, based on pre-procedural risk assessment, might help in guiding the choices of the physicians, in terms of treatment and cost-effectiveness.

Thereby, the assessment of sarcopenia could become an additional step before the procedure in elderly patients, as the alone did not correlate with ERCP safety in the literature [33]. At present, CT-scan assessment of the skeletal muscle area (SMA, cm^2^) and skeletal muscle index (SMI, cm^2^/m^2^) at the L3 level are widely used as objective surrogate markers of sarcopenia [11,12], and specific diagnostic cutoffs have been recognized for older patients [13].

In our study, the overall prevalence of sarcopenia was 11.7%, in line with what has been reported in the literature for elderly patients [34]. In contrast, the higher short- and medium-term mortality rates of 7.8% and 16.8%, respectively, likely reflect the increased risk of mortality in elderly patients and the relatively high prevalence of hepatobiliary neoplasia. For the same reason, the rates of ERCP-related complications and of cardiopulmonary complications were 14.8% and 3.5%, slightly higher than those reported in the literature.

Interestingly, sarcopenia was the only factor associated with short-term mortality, while long-term mortality was also associated with a higher ASA score and CCI, as well as the presence of cancer.

Possible explanations for the prolonged post-operative hospitalization duration in sarcopenic patients might be the higher rate of post-procedural complications and the lower reserve capacity for early recovery.

It is not known if the recognition of sarcopenia in patients ongoing ERCP could influence the clinical decision making processes by serving as an objective marker for risk stratification along with other well-known patient and procedural risk factors. The decision of performing or avoiding a therapeutic intervention exposes physicians to a lot of ethical concerns. Population in the western world is constantly becoming older, people life longer with a wide spectrum of diseases and the presence of elderly patients needing interventional hepatobiliary endoscopy has been increasing. We need more evidences to assess the risk of adverse events and death in those patients. CT scan measurement of sarcopenia is a relatively simple objective tool and could help the clinicians in taking decision more wisely. In case of benign diseases with no urgency patient optimization strategies (i.e. nutritional supplementation) could be considered before the procedure. In case benign urgent or malignant settings, the awareness of the higher risk ok mortality could help an open discussion with the patient and the family, improving the informed consent. The awareness of sarcopenia could suggest to manage the patient in intensive care units. We also recognize that this study has some limitations. The retrospective single-center design and the small number of enrolled patients limits study’s generalizability. Indeed, the subgroup analysis performed on sarcopenic patients included only 30 subjects. This requires validation of the results with larger multicenter cohort studies. In addition, the relatively short follow-up time did not allow the assessment of long-term outcomes, and the absence of a longitudinal assessment of sarcopenia did not allow us to evaluate how its dynamic progression or regression over time might impact with ERCP outcomes.

Short- and long-term mortality rates were retrieved from the hospital electronic health records, hence potentially underestimating the effective rates. Moreover, muscle function assessment (i.e. handgrip strength) to assess sarcopenia was not performed, as well as the measurements of several biochemical parameters that correlate with frailty, in particular albumin, whose association with ERCP-outcomes is still little investigated.

Indeed, even if hypoalbuminemia has been strongly associated with increased surgical morbidity and mortality, evidence on ERCP-related complications did not include it as a possible risk factor [35]. Most patients undergoing ERCP have ongoing acute inflammatory processes potentially leading to hypoalbuminemia secondary to an increased capillary permeability and consequent extravasation of serum albumin into the interstitial space [36]. Last, the exclusion of patients without pre-procedural CT scans might have introduced selection bias, as these individuals may differ in clinical characteristics or outcomes from those undergoing CT. Further studies should be implemented to address these questions.

However, our study also has several strengths. It is the first one assessing the impact of sarcopenia in the context of ERCP, even if in a retrospective series. Sarcopenia assessment does not require any additional imaging or tests other than CT scans, that is frequently performed in most elderly patients with jaundice, to exclude malignancy. This enables to retrieval of the information required to measure sarcopenia without any additional tests or costs. Moreover, the median time from CT scan to ERCP in our study was three days. This made the CT measurements highly representative of the real muscle mass of the enrolled patients, minimizing the possible bias of the data collection. So far, the most important outcome of any invasive procedure is the mortality, especially in elderly patients.

If the results of our study are confirmed in a larger prospective series, the assessment of sarcopenia could become an easy tool to stratify the patients into two big classes of risk, thus impacting safety in the ERCP setting.

## 5. Conclusions

This study suggests that sarcopenia can be considered a possible predictor of short- and medium-term mortality and prolonged post-procedural hospitalization in elderly patients undergoing ERCP. These findings underscore the importance of pre-procedural risk stratification, incorporating objective measures such as sarcopenia assessment alongside established clinical indices like the ASA score and CCI. Identifying sarcopenia may help guide clinical decision-making, enabling tailored peri-procedural strategies to improve patient outcomes.

## Figures and Tables

**Table 1:** Baseline demographic patient characteristics in the sarcopenic and non-sarcopenic cohort.

	Variable	Sarcopenic Group (30)	Non-Sarcopenic Group (226)	*p*-Value
Patient characteristics	Age (years), mean (SD)	83.9 (5.08)	82.2 (4.69)	ns
	Sex (male), *n* (%)	20 (66)	114 (50)	ns
	Waist circumference (cm), median (IQR)	105 (93.4–116.1)	101.1 (93.6–110.2)	ns
	ASA, *n* (%)			
	1–2	11 (36.6)	112 (43.7)	ns
	3–4	19 (65.5)	114 (50.7)	ns
	CCI, mean (SD)	7.23 (2.05)	6.63 (2.52)	ns

ASA: American Society of Anesthesiologists Score; CCI: Charlson Comorbidity Index.

**Table 2:** Frequency of overall complications in sarcopenic and non-sarcopenic patients.

	Sarcopenic	Non-Sarcopenic	*p*-Value
Postprocedural complications, *n* (%)	9 (30)	40 (17.7)	ns
Postprocedural mortality, *n* (%)			
30 days	7 (23.3)	14 (6.2)	0.001
12 months	12 (40)	31 (13.7)	0.003
Postprocedural hospitalization duration (days), median (IQR)	11 (7–17)	7 (4–12)	0.003

**Table 3:** Demographic and clinical variables associated with post-ERCP mortality at 30 days and at 1 year as well as overall post-ERCP complications. Univariate and multivariate logistic regression analysis. In the multivariate model, only the variables with *p* < 0.05 are reported. Bold values denote statistical significance.

Post-Ercp 30-Days Mortality				
Parameter	Univariate Analysis		Multivariate Analysis	
	OR (95% CI)	*p* Value	OR (95% CI)	*p* Value
Sarcopenia				
No	1.0 (ref)		1.0 (ref)	
Yes	4.61 (1.69–12.58)	0.03	3.45 (1.13–10.61)	0.03
Age (years)	1.0 (0.91–1.10)	ns	-	-
CCI, mean	1.09 (0.95–1.26)	ns	-	-
ASA score				
1–2	1.0 (ref)			
3–4	2.44 (0.91–6.50)	ns	-	-
Procedure complexity (ASGE)				
1–2	1.0 (ref)			
3–4	0.60 (0.13–2.70)	ns	-	-
Cancer				
No	1.0 (ref)			
Yes	2.44 (0.98–6.10)	ns	-	-
Post-Ercp 1-Year Mortality				
Sarcopenia				
No	1.0 (ref)			
Yes	4.19 (1.84–9.55)	<0.001	3.87 (1.55–9.60)	0.004
Age (years)	1.0 (0.94–1.08)	ns	-	-
CCI, mean	1.69 (0.99–2.87)	0.03	1.36 (1.16–1.59)	<0.001
ASA score			-	-
1–2	1.0 (ref)			
3–4	3.16 (1.51–6.60)	0.002	3.18 (1.40–7.25)	0.006
Procedure complexity (ASGE)				
1–2	1.0 (ref)			
3–4	0.56 (0.18–1.67)	ns	-	-
Cancer				
No	1.0 (ref)			
Yes	2.94 (1.48–5.83)	0.002	1.84 (0.83–4.05)	0.007
Post-Ercp Complications				
Sarcopenia				
No	1.0 (ref)			
Yes	2.01 (0.81–4.93)	ns	-	-
Age (years)	0.95 (0.88–1.02)	ns	-	-
CCI, mean	0.97 (0.84–1.12)	ns	-	-
ASA score				
1–2	1.0 (ref)			
3–4	0.70 (0.36–1.35)	ns	-	-
Procedure complexity (ASGE)				
1–2	1.0 (ref)			
3–4	0.47 (0.15–1.43)	ns	-	-
Cancer				
No	1.0 (ref)			
Yes	1.72 (0.83–3.57)	ns	-	-

ASA: American Society of Anesthesiologists Score; CCI: Charlson Comorbidity Index.

## Data Availability

The data presented in this study are available on request from the corresponding author due to the presence of sensitive data from the patients.

## Supplementary Materials

The following supporting information can be downloaded at: https://www.mdpi.com/article/10.3390/life15010021/s1, Table S1: Demoghraphic and clinical variables associated to post-ERCP mortality at 12 in non-cancer patients. Univariate and Multivariate analysis.

## Appendix A

**Table A1:** Clinical Indications for ERCP in the study cohort.

ERCP-Indication	N (%)
Hepatobiliary cancer	60 (23.4)
Choledocolithiasis	69 (26.9)
Acute pancreatitis	16 (6.3)
Cholecystitis/cholangitis	57 (22.3)
Surgical complications	8 (3.1)
Other	46 (17.9)

## References

[1] ASGE Guidelines for Clinical Application (1999). The role of ERCP in diseases of the biliary tract and pancreas. American Society for Gastrointestinal Endoscopy. Gastrointest. Endosc..

[2] Dumonceau J.-M., Kapral C., Aabakken L., Papanikolaou I.S., Tringali A., Vanbiervliet G., Beyna T., Dinis-Ribeiro M., Hritz I., Mariani A. (2020). ERCP-related adverse events: European Society of Gastrointestinal Endoscopy (ESGE) Guideline. Endoscopy.

[3] Bodger K., Bowering K., Sarkar S., Thompson E., Pearson M.G. (2011). All-cause mortality after first ERCP in England: Clinically guided analysis of hospital episode statistics with linkage to registry of death. Gastrointest. Endosc..

[4] Cotton P.B., Lehman G., Vennes J., Geenen J.E., Russell R.C., Meyers W.C., Liguory C., Nickl N. (1991). Endoscopic sphincterotomy complications and their management: An attempt at consensus. Gastrointest. Endosc..

[5] Andriulli A., Loperfido S., Napolitano G., Niro G., Valvano M.R., Spirito F., Pilotto A., Forlano R. (2007). Incidence rates of post-ERCP complications: A systematic survey of prospective studies. Am. J. Gastroenterol..

[6] Shavakhi A., Zobeiri M., Khodadoostan M., Zobeiri M.J., Shavakhi A. (2023). Risk factors for ERCP-related complications and what is the specific role of ASGE grading system. J. Res. Med. Sci..

[7] Katsinelos P., Lazaraki G., Chatzimavroudis G., Gkagkalis S., Vasiliadis I., Papaeuthimiou A., Terzoudis S., Pilpilidis I., Zavos C., Kountouras J. (2014). Risk factors for therapeutic ERCP-related complications: An analysis of 2,715 cases performed by a single endoscopist. Ann. Gastroenterol..

[8] Chawla S., Willingham F.F. (2014). Cardiopulmonary complications of endoscopic retrograde cholangiopancreatography. Tech. Gastrointest. Endosc..

[9] Daabiss M. (2011). American Society of Anaesthesiologists physical status classification. Indian J. Anaesth..

[10] Cotton P.B., Eisen G., Romagnuolo J., Vargo J., Baron T., Tarnasky P., Schutz S., Jacobson B., Bott C., Petersen B. (2011). Grading the complexity of endoscopic procedures: Results of an ASGE working party. Gastrointest. Endosc..

[11] Sahar N., La Selva D., Gluck M., Gan S.I., Irani S., Larsen M., Ross A.S., Kozarek R.A. (2019). The ASGE grading system for ERCP can predict success and complication rates in a tertiary referral hospital. Surg. Endosc..

[12] Cruz-Jentoft A.J., Bahat G., Bauer J., Boirie Y., Bruyère O., Cederholm T., Cooper C., Landi F., Rolland Y., Sayer A.A. (2019). Sarcopenia: Revised European consensus on definition and diagnosis. Age Ageing.

[13] Erős A., Soós A., Hegyi P., Szakács Z., Benke M., Szűcs Á., Hartmann P., Erőss B., Sarlós P. (2020). Sarcopenia as an independent predictor of the surgical outcomes of patients with inflammatory bowel disease: A meta-analysis. Surg. Today.

[14] Hajibandeh S., Hajibandeh S., Jarvis R., Bhogal T., Dalmia S. (2019). Meta-analysis of the effect of sarcopenia in predicting postoperative mortality in emergency and elective abdominal surgery. Surg..

[15] Park B., Bhat S., Wells C.I., Barazanchi A.W.H., Hill A.G., MacCormick A.D. (2022). Short- and long-term impact of sarcopenia on outcomes after emergency laparotomy: A systematic review and meta-analysis. Surgery.

[16] Jones K., Gordon-Weeks A., Coleman C., Silva M. (2017). Radiologically Determined Sarcopenia Predicts Morbidity and Mortality Following Abdominal Surgery: A Systematic Review and Meta-Analysis. World J. Surg..

[17] Rezende I.F.B., Conceição-Machado M.E.P., Souza V.S., Santos E.M.D., Silva L.R. (2020). Sarcopenia in children and adolescents with chronic liver disease. J. Pediatr..

[18] Mulinacci G., Pirola L., Gandola D., Ippolito D., Viganò C., Laffusa A., Gallo C., Invernizzi P., Danese S., Massironi S. (2024). Ultrasound muscle assessment for sarcopenia detection in inflammatory bowel disease: A prospective study. United Eur. Gastroenterol. J..

[19] Quando si Diventa “Anziani”?. https://www.sigg.it/news-geriatria/quando-si-diventa-anziani/.

[20] Van Vugt J.L.A., Levolger S., Gharbharan A., Koek M., Niessen W.J., Burger J.W.A., Willemsen S.P., De Bruin R.W.F., IJzermans J.N.M. (2017). A comparative study of software programmes for cross-sectional skeletal muscle and adipose tissue measurements on abdominal computed tomography scans of rectal cancer patients. J. Cachexia Sarcopenia Muscle.

[21] Tagliafico A.S., Bignotti B., Torri L., Rossi F. (2022). Sarcopenia: How to measure, when and why. Radiol. Med..

[22] van der Werf A., Langius J.A.E., de van der Schueren M.A.E., Nurmohamed S.A., van der Pant K.A.M.I., Blauwhoff-Buskermolen S., Wierdsma N.J. (2018). Percentiles for skeletal muscle index, area and radiation attenuation based on computed tomography imaging in a healthy Caucasian population. Eur. J. Clin. Nutr..

[23] Lin H.-S., Watts J.N., Peel N.M., Hubbard R.E. (2016). Frailty and post-operative outcomes in older surgical patients: A systematic review. BMC Geriatr..

[24] Makary M.A., Segev D.L., Pronovost P.J., Syin D., Bandeen-Roche K., Patel P., Takenaga R., Devgan L., Holzmueller C.G., Tian J. (2010). Frailty as a predictor of surgical outcomes in older patients. J. Am. Coll. Surg..

[25] Sasegbon A., Weerasinghe P., Lal S. (2023). The relationships between sarcopenia, frailty, bioelectrical impedance analysis, and anthropometry in patients with type two intestinal failure. Clin. Nutr. ESPEN.

[26] Chen F., Chi J., Liu Y., Fan L., Hu K. (2022). Impact of preoperative sarcopenia on postoperative complications and prognosis of gastric cancer resection: A meta-analysis of cohort studies. Arch. Gerontol. Geriatr..

[27] Lutz C.T., Quinn L.S. (2012). Sarcopenia, obesity, and natural killer cell immune senescence in aging: Altered cytokine levels as a common mechanism. Aging.

[28] Saucy F., Probst H., Hungerbühler J., Maufroy C., Ricco J.-B. (2024). Impact of Frailty and Sarcopenia on Thirty-Day and Long-Term Mortality in Patients Undergoing Elective Endovascular Aortic Aneurysm Repair: A Systematic Review and Meta-Analysis. J. Clin. Med..

[29] Salman M.A., Omar H.S.E., Mikhail H.M.S., Tourky M., El-Ghobary M., Elkassar H., Omar M.G., Matter M., Elbasiouny A.M., Farag A.M. (2020). Sarcopenia increases 1-year mortality after surgical resection of hepatocellular carcinoma. ANZ J. Surg..

[30] Knoedler S., Schliermann R., Knoedler L., Wu M., Hansen F.J., Matar D.Y., Obed D., Vervoort D., Haug V., Hundeshagen G. (2023). Impact of sarcopenia on outcomes in surgical patients: A systematic review and meta-analysis. Int. J. Surg. Lond. Engl..

[31] Koh F.H., Chua J.M., Tan J.L., Foo F.-J., Tan W.J., Sivarajah S.S., Ho L.M.L., Teh B.-T., Chew M.-H. (2021). Paradigm shift in gastrointestinal surgery—Combating sarcopenia with prehabilitation: Multimodal review of clinical and scientific data. World J. Gastrointest. Surg..

[32] Tomassini S., Abbasciano R., Murphy G.J. (2021). Interventions to prevent and treat sarcopenia in a surgical population: A systematic review and meta-analysis. BJS Open.

[33] Colmenero Gargari A.E., Melgar Somoza F.E., Vera J., Micames C.G. (2023). ERCP in patients over 90 years old: Safety and efficacy comparison with a younger cohort. Endosc. Int. Open.

[34] Yuan S., Larsson S.C. (2023). Epidemiology of sarcopenia: Prevalence, risk factors, and consequences. Metabolism.

[35] Makmun D., Abdullah M., Syam A.F., Fauzi A. (2015). PostERCP pancreatitis and its related factors: A prospective study in Cipto Mangunkusumo National General Hospital. J. Dig. Endosc..

[36] Soeters P.B., Wolfe R.R., Shenkin A. (2019). Hypoalbuminemia: Pathogenesis and Clinical Significance. J. Parenter. Enter. Nutr..

