# Supplementary material for: Sarcopenia as a Predictor of Mortality in a Cohort of Elderly Patients Undergoing Endoscopic Retrograde Cholangiopancreatography
Source: Life (Basel). 2024 Dec 28;15(1):21. doi: 10.3390/life15010021 (PMC11767023; doi:10.3390/life15010021)
Supplement: Supplementary file 1 [file life-15-00021-s001.zip › life-3362801-supplementary.pdf]

POST-ERCP MORTALITY				
12 MONTHS				
**Parameter**	**Univariate analysis**		**Multivariate analysis**	
	**OR (95% CI)**	**P value**	**OR (95% CI)**	**P value**
Age (years)	1.02 (0.93-1.12)	ns	-	-
Sarcopenia				
No	1.0 (ref)			
Yes	4.38 (1.35-14.18)	0.014	3.62 (1.05-12.52)	0.04
CCI, mean	1.28 (1.07-1.54)	0.007	1.26 (1.03-1.55)	0.02
ASA			-	-
1-2	1.0 (ref)			
3-4	4.30 (1.39-13.33)	0.011	3.48 (1.07-11.24)	0.04
ASGE				
1-2	1.0 (ref)			
3-4	0.44 (0.09-1.98)	ns	-	-

Supplementary Table S1. Demoghraphic and clinical variables associated to post-ERCP mortality at 12 in non-cancer patients. Univariate and Multivariate analysis.
